# Interocular Differences in Corneal Asphericity Are Associated with Stereopsis in Myopic Astigmatism Candidates for Laser Vision Correction

**DOI:** 10.1155/joph/6398823

**Published:** 2026-06-04

**Authors:** Alireza Peyman, Mahsa Pourmahdi-Boroujeni, Pegah Noorshragh, Faezeh Fayaz, Mohsen Pourazizi, Emad Salimian

**Affiliations:** ^1^ Isfahan Eye Research Center, Department of Ophthalmology, Isfahan University of Medical Sciences, Isfahan, Iran, mui.ac.ir; ^2^ Isfahan Eye Research Center, Isfahan University of Medical Sciences, Isfahan, Iran, mui.ac.ir; ^3^ Cumming School of Medicine, Department of Community Health Sciences, University of Calgary, Calgary, Alberta, Canada, ucalgary.ca; ^4^ Department of Optometry, School of Rehabilitation Sciences, Rehabilitation Research Center, Iran University of Medical Sciences, Tehran, Iran, iums.ac.ir; ^5^ Student Research Committee, Isfahan University of Medical Sciences, Isfahan, Iran, mui.ac.ir

**Keywords:** asphericity, corneal topography, refractive surgical procedures, stereoacuity, vision disparity

## Abstract

**Purpose:**

To investigate the relationship between stereoacuity and corneal topographic parameters in patients with compound myopic astigmatism who are candidates for laser vision correction (LVC).

**Methods:**

In this analytical cross‐sectional study, LVC candidates aged 18 years or older with compound myopic astigmatism and corrected distance visual acuity of 20/25 or better were enrolled. Stereoacuity was assessed using the stereo fly test, and corneal topography parameters were measured with Scheimpflug imaging (Pentacam HR). The interocular differences in each topographic parameter were calculated. Pearson correlation and multiple linear regression were used to evaluate the associations between stereoacuity and interocular differences in topographic parameters.

**Results:**

The mean (SD) age of participants was 29.84 (7.39) years, and the average stereoacuity was 45.52 (13.75) arc/sec (log‐transformed stereoacuity: 1.65 (0.09)). A statistically significant correlation was found between interocular differences in corneal asphericity (Q value) and log‐transformed stereoacuity (*r* = 0.27, *p* = 0.011). No significant associations were observed between stereoacuity and other corneal topographic parameters. Also, the interocular difference in corneal asphericity was the strongest predictor of stereoacuity (*β* = 0.315, 95% CI: [0.406, 1.820], *p* = 0.002).

**Conclusion:**

This study identified a significant relationship between interocular corneal asphericity difference and stereoacuity in patients with compound myopic astigmatism.

## 1. Introduction

Stereopsis, as a component of binocular vision, arises from the slight variations in the images seen by each eye due to their horizontal separation, resulting in binocular disparity [1]. It enables humans to assess the distance and depth of objects to develop precise motor skills and coordination, such as driving, playing sports, and performing surgery [2, 3]. It has been utilized in preemployment screenings for critical roles, such as piloting or surgery, highlighting the significance of stereopsis in modern society [4]. Stereoacuity can be impacted by various factors, including age, optical blur, interocular differences, refractive errors, anisometropia, aniseikonia, reduced contrast sensitivity, strabismus, and amblyopia [5, 6].

In a normal population with well‐balanced and focused optics, stereopsis is typically within the normal range [7]. However, in individuals with keratoconus, stereoacuity is significantly reduced, primarily due to interocular differences in refractive errors and loss of contrast sensitivity [8, 9]. Similarly, in cases of high anisometropia (greater than 6.0 diopters), stereoacuity is markedly compromised compared to that in individuals with isometropia or emmetropia, likely as a result of foveal suppression in the more defocused eye [10].

While numerous studies have explored the relationship between stereopsis and factors such as age, ocular disorders, visual acuity, and the outcomes of refractive surgeries [5, 11–16], there is a lack of data on the association between stereopsis and corneal topography parameters in patients with compound myopic astigmatism. Corneal topography is broadly used in the screening of keratoconus, contact lens fitting, assessment of ectasia progression, calculation of cataract and intraocular lens power, measurement of surgical outcomes in refractive surgery, and corneal transplants [17, 18].

In this study, we investigated the relationship between stereoacuity and corneal topography parameters in patients with compound myopic astigmatism undergoing laser vision correction (LVC).

## 2. Methods and Materials

### 2.1. Study Design and Setting

This analytical cross‐sectional study was conducted at Feiz Ophthalmology Hospital in Isfahan, Iran, and adhered to the ethical principles outlined in the Declaration of Helsinki. All patients were provided with a clear explanation of the study’s objectives, and their informed consent was obtained. The study’s methodology was affirmed by the institutional review board and ethical committee of Isfahan University of Medical Sciences (IR.MUI.MED.REC.1402.153).

### 2.2. Participants

This study was performed on patients with compound myopic astigmatism who were candidates for LVC. The inclusion criteria for the study were as follows: (1) age of 18 years or older, (2) corrected distance visual acuity (CDVA) of 20/25 or better, (3) central corneal thickness of at least 490 microns (μm), (4) spherical equivalent (SE) refraction ranging from −0.25 to −6.0 diopters (D), and (5) documented stable refraction (refractive change of less than 0.5 diopters for at least 1 year prior to surgery) [19–21].

The exclusion criteria were a history of ocular surgeries or eye trauma, systemic diseases with ocular impacts (e.g., diabetes or collagen vascular diseases), ocular diseases (e.g., cataracts and keratoconus), use of systemic or ocular medications affecting binocular or accommodative vision, presence of binocular anomalies (e.g., strabismus or decompensated heterophoria), individuals with a preoperative stereopsis test worse than 480 arc/sec, monocular individuals, amblyopia, individuals whose pupil size falls outside the normal range (7–8 mm in mesopic conditions), individuals with unequal pupil sizes, abnormal pupillary distance (PD) (outside the 54–72 mm range), anisometropia ≥ 1 diopter, and any other condition affecting depth perception.

### 2.3. Ophthalmic Assessment

Visual acuity was determined using the Snellen acuity chart (Mediz LED chart, Smart LC 13, Korea). Refraction was assessed using manifest and cycloplegic methods. A slit‐lamp biomicroscopy examination (Topcon, SL‐D301, Japan) was used to inspect the anterior segment of the eye directly. Stereoacuity (arc/sec) was measured using the stereo fly test (Stereo Optical, USA), which utilizes a booklet with polarized test images at a 40‐cm testing distance and requires the use of cross‐polarized lenses in a quiet, shaded room with standard daylight (600 lux) and heat (22°C). The subjects wore specialized eyeglasses during the test [22, 23].

The corneal evaluation was conducted using Scheimpflug corneal tomography (Pentacam HR, Oculus, Germany), which encompassed measurements of pupil size, average keratometry, corneal simulated keratometry (simK), keratometric astigmatism (K flat, K steep, and axis), total corneal astigmatism, central corneal thickness, corneal asphericity (*Q* values), and chord mu.

Additionally, ocular residual astigmatism (ORA), defined as the difference between refractive and corneal astigmatism, was calculated.

### 2.4. Statistical Analyses

To improve the accuracy of the statistical analysis, the stereoacuity values were converted to the logarithm of the seconds of the arc. Qualitative variables were reported as frequencies and percentages, while quantitative variables were reported as means (standard deviations) and medians. The normality of the quantitative variables was evaluated using the Kolmogorov–Smirnov test. The Pearson correlation tests were employed to evaluate the association between corneal topographic parameters and stereoacuity. Multiple linear regression analysis was performed to identify predictors of log‐transformed stereoacuity. For each topographic parameter, the interocular differences (i.e., the absolute differences between the two eyes) were used in the correlation analysis. All statistical analyses were conducted using the SPSS software (Version 25, Chicago, IL, USA) [24]. Statistical significance was set at *p* value < 0.05.

## 3. Results

Ninety‐six patients with compound myopic astigmatism, including 70 females (72.9%) and having a mean age of 29.84 (7.39), were enrolled. The mean PD was 62.29 (3.51) mm. The mean stereoacuity was 45.52 (13.75) arc/sec, with a log‐transformed stereoacuity value of 1.65 (0.09). The mean SE was −3.55 (1.69) D. The detailed baseline characteristics of the patients, including visual acuity, keratometry values, pupil size, corneal asphericity, central corneal thickness, and ORA for each eye, are presented in Table 1 (Table 1).

**TABLE 1:** Baseline characteristics of the patients enrolled in the study.

Variable	OD	OS	Difference
Age, years	29.84 (7.39)		
Sex (male) *n* (%)	26 (27.1)		
PD, mm	62.29 (3.51)		
Stereoacuity	45.52 (13.75)		
LogStereoacuity	1.65 (0.09)		
SE	−3.55 (1.69)		
Sphere	−2.97 (1.75)	−2.93 (1.83)	0.47 (0.64)
Cylinder	−1.16 (1.09)	−1.19 (1.21)	0.50 (0.69)
LogUCVA	0.70 (0.38)	0.96 (0.39)	0.23 (0.21)
LogCDVA	0.0 (0.005)	0.0 (0.006)	0.0 (0.00)
Accommodation	11.76 (4.92)	11.79 (5.05)	0.037 (1.97)
$\sqrt{{Chord\;x}^{2}+{Chord\;y}^{2}}$	0.024 (0.02)	0.023 (0.026)	0.0 (0.00)
Pupil size	4.32 (1.41)	4.29 (1.39)	0.38 (0.38)
Steep K	44.6 (1.91)	44.65 (1.94)	0.35 (0.31)
Flat K	43.18 (1.78)	43.14 (1.78)	0.30 (0.31)
Corneal asphericity (*Q* value)	−0.25 (0.98)	−0.26 (0.10)	0.04 (0.04)
CCT	539.05 (30.77)	540.20 (30.43)	4.57 (4.46)
ORA	0.81 (0.40)	0.77 (0.31)	0.27 (0.25)

Abbreviations: CCT, central corneal thickness; CDVA, corrected distance visual acuity; K, keratometry; ORA, ocular residual astigmatism; PD, pupillary distance; SD, standard deviation; SE, spherical equivalent; UCVA, uncorrected visual acuity.

The Pearson correlation analysis assessed the relationship between the log‐transformed stereoacuity and various corneal topographic parameters. The correlation results are presented in Table 2. The results showed that the interocular difference in the corneal asphericity had a statistically significant correlation with the log‐transformed stereoacuity (Pearson correlation coefficient = 0.27, *p* value = 0.011). However, other corneal topographic parameters, including differences in spherical and cylindrical refraction, chord values, PD, keratometry values, corneal thickness, and ORA, showed no statistically significant association with the log‐transformed stereoacuity (all *p* values > 0.05) (Table 2).

**TABLE 2:** Correlations between log‐transformed stereoacuity and topography findings.

Variables	Pearson correlation	*P* value
Diff sphere	0.12	0.238
Diff cylinder	0.58	0.572
Diff accommodation	0.87	0.400
Diff chord x	−0.07	0.487
Diff chord y	0.11	0.275
Diff pupil size	0.01	0.950
Diff steep K	−0.01	0.909
Diff flat K	0.08	0.419
Diff CCT	−0.15	0.151
Diff ORA	0.03	0.754
Diff Q‐value	0.27	**0.011** ^**∗**^

*Note:* Bold value indicates statistical significance (*p* < 0.05).

Abbreviations: CCT, central corneal thickness; K, keratometry; ORA, ocular residual astigmatism.

Multiple linear regression was performed to identify predictors of log‐transformed stereoacuity in patients. The overall model was statistically significant, *F* (5, 82) = 3.913, *p* = 0.003, explaining 19.3% of the variance in stereoacuity (*R* ^2^ = 0.193, adjusted *R* ^2^ = 0.144).

Among the predictor variables, interocular differences in corneal asphericity emerged as the strongest and most significant predictor (*β* = 0.315, *p* = 0.002). The unstandardized coefficient indicated that for each unit, the interocular corneal asphericity difference increased, the log stereoacuity increased by 1.113 units. Sex was also a significant predictor (*β* = −0.340, *p* = 0.007), with males showing better stereoacuity compared to females when controlling for other variables. The unstandardized coefficient indicates that males had log stereoacuity values that were 0.071 units lower than females. However, analyses of parameters including age, PD, and CCT were statistically insignificant (all *p* values > 0.05) (Table 3).

**TABLE 3:** Multiple linear regression analysis predicting log stereoacuity.

Variable	*B*	SE	*β*	*t*	*p* value	95% CI
(Constant)	1.397	0.190	—	7.347	< 0.001	[1.020, 1.774]
Age	−0.002	0.001	−0.131	−1.281	0.204	[−0.005, 0.001]
Sex	−0.071	0.025	−0.340	−2.789	0.007	[−0.121, −0.020]
Pupillary distance	0.006	0.003	0.211	1.783	0.078	[−0.001, 0.013]
Diff CCT	−0.004	0.002	−0.176	−1.711	0.091	[−0.008, 0.001]
Diff Q‐value	1.113	0.356	0.315	3.126	0.002	[0.406, 1.820]

*Note:* *B* = unstandardized coefficient; β = standardized coefficient, Sex coded as male = 1, female = 0; Diff Q‐value = interocular difference in corneal asphericity.

Abbreviations: CCT = central corneal thickness, SE = standard error.

## 4. Discussion

This study investigated the correlation between stereoacuity and corneal topographic findings in compound myopic astigmatism patients. Our findings demonstrated that among the corneal topographic parameters, the corneal asphericity difference had a statistically significant correlation with stereoacuity; however, other parameters, including PD, keratometry values, central corneal thickness, and ORA, did not. In the regression analyses, corneal asphericity difference was the strongest predictor of stereoacuity. Sex was also a significant predictor, with males showing better stereoacuity when controlling for other variables. Other parameters, including age, PD, and CCT, were statistically insignificant in the regression model.

This correlation between stereoacuity and asphericity demonstrates that variations in corneal shape might impact binocular disparity, aligning with previous studies [25, 26]. This correlation can be explained by the complex interplay between corneal shape, optical performance, and binocular vision. When there is a notable difference in optical quality between the two eyes caused by variations in corneal shape, the visual system might find it challenging to effectively combine these differing images, which could affect stereoacuity [7].

Corneal asphericity affects the distribution of higher‐order aberrations (HOAs), especially spherical aberrations [27, 28]. HOAs have been shown to influence visual acuity, contrast sensitivity, and retinal image quality [29–31]. Additionally, previous studies indicated that HOAs and interocular differences in HOAs can influence binocular vision and stereopsis [32, 33]. The noted correlation in our study likely results from asymmetries in optical quality and HOAs between the eyes caused by interocular differences in corneal asphericity [32, 34].

Keratometry values indicating corneal curvature have been shown to influence HOAs and visual outcomes in refractive surgery [35, 36]. Based on our results, the overall corneal curvature in a normal cornea may not directly affect depth perception. However, excessive deviations in keratometry, such as those observed in keratoconus, can certainly affect stereopsis [9, 37, 38].

Central corneal thickness is an important factor in refractive surgery and a key predictor for postoperative complications such as ectasia. Nevertheless, our findings suggest that it is not associated with stereoacuity. It could be interpreted that within the normal range found in LVC candidates in our study, it does not necessarily have a significant linear association with stereoacuity [39, 40].

PD is a fundamental factor in binocular vision and has been considered crucial in stereopsis as it affects the extent of binocular disparity [41]. Nonetheless, our results indicate no notable relationship between PD and stereoacuity. It implies that in the normal range of PDs observed in LVC candidates, this factor may not be a significant determinant of depth perception. This result is consistent with the previous study by Eom et al., showing that stereoacuity remained constant across a range of normal PDs [42].

ORA influences visual performance, contrast sensitivity, visual acuity, and refractive surgery outcomes [43–45]. The current study showed that ORA has an insignificant association with stereoacuity. This contrasts with previous studies demonstrating a relation between astigmatism and stereoacuity [46, 47]. This observation could be a result of a low ORA measure for each eye (< 1D). It showed that within the normal range for ORA found in LVC candidates, it does not affect stereoacuity significantly.

Future research should explore the longitudinal effects of LVC on stereoacuity, particularly regarding induced changes in corneal asphericity. It would be beneficial to determine if comparable relationships exist in individuals considering any types of laser refractive procedures, such as PRK, LASIK, or small incision lenticule extraction (SMILE). Additionally, research with precise assessments of other aspects of visual function, such as contrast sensitivity and wavefront analyses, may provide a better understanding of stereopsis. Additionally, in the multiple linear regression model, males demonstrated better stereoacuity than females, suggesting that the sex effect should be further studied in future research.

However, our study had limitations that future research could address. The cross‐sectional design limited the determination of causal relationships and longitudinal effects between the variables. Although the sample size was sufficient for identifying significant correlations, it may hinder the generalizability of the findings to larger populations. Additionally, the emphasis on LVC candidates restricts the applicability of this study to other refractive surgery methods or nonsurgical populations. Utilizing a singular stereoacuity test (the stereo fly test and not other tests like randot circles) may not fully reflect the complete spectrum of stereoscopic vision. Future research could benefit from using various methods to evaluate stereoacuity.

## 5. Conclusion

In conclusion, our study revealed a significant linear correlation between interocular differences in corneal asphericity and stereoacuity among all evaluated topographic parameters within normal clinical ranges in compound myopic astigmatism patients, with the corneal asphericity difference being the strongest predictor of stereoacuity. This underscores the need to implement corneal asphericity assessments in preoperative evaluations and consider binocular vision outcomes when planning more customized and efficient refractive surgery to achieve better vision correction and higher patient satisfaction in LVC.

**Nomenclature**
PRK: Photorefractive keratectomy
LVC: Laser vision correction
CDVA: Corrected distance visual acuity
UCVA: Uncorrected visual acuity
SE: Spherical equivalent
D: Diopters
μm: Microns
PD: Pupillary distance
OD: Oculus dexter (right eye)
OS: Oculus sinister (left eye)
K: Keratometry
CCT: Central corneal thickness
ORA: Ocular residual astigmatism
HOAs: Higher‐order aberrations
SMILE: Small incision lenticule extraction
B: Unstandardized coefficient
β: Standardized coefficient

## Author Contributions

A.P and M.P provided the concept of the study and contributed to designing the study. F.F and E.S assisted in data gathering. P.N and M.P assisted in data analyzing. E.S and M.P.B. assisted in the preparation of the first draft of the manuscript. A.P, M.P, F.F, and P.N revised the manuscript critically for important intellectual content.

## Funding

None of the authors has any financial disclosures. The authors of this article received no financial support for their research, drafting, or publishing.

## Disclosure

All authors have read the final version and approved the content of the manuscript to be published and confirmed the accuracy or integrity of any parts of the work.

We declare that none of the authors are employed by a government agency that has a primary function other than research and/or education. None of the authors have an official representative on behalf of the government.

## Conflicts of Interest

The authors declare no conflicts of interest.

## Data Availability

The data used to support the findings of this study are available from the corresponding author upon written request.

## References

[1] Filippini H. R. and Banks M. S. , Limits of Stereopsis Explained by Local Cross-Correlation, Journal of Vision. (2009) 9, no. 1, 8.1–18, 10.1167/9.1.8. 19271878 PMC2940423

[2] Mazyn L. I. , Lenoir M. , Montagne G. , and Savelsbergh G. J. , The Contribution of Stereo Vision to One-Handed Catching, Experimental Brain Research. (2004) 157, no. 3, 383–390, 10.1007/s00221-004-1926-x. 15221161

[3] Wong N. W. , Stokes J. , Foss A. J. , and McGraw P. V. , Should There be a Visual Standard for Ophthalmologists and Other Surgeons?, Postgraduate Medical Journal. (2010) 86, no. 1016, 354–358, 10.1136/pgmj.2009.091371. 20547602

[4] Donate D. , Denis P. , and Burillon C. , [Prospective Study of Contrast Sensitivity and Visual Effects After LASIK], Journal Français d’Ophtalmologie. (2005) 28, no. 10, 1070–1075, 10.1016/s0181-5512(05)81140-4. 16395199

[5] Costa M. F. , Moreira S. M. , Hamer R. D. , and Ventura D. F. , Effects of Age and Optical Blur on Real Depth Stereoacuity, Ophthalmic and Physiological Optics. (2010) 30, no. 5, 660–666, 10.1111/j.1475-1313.2010.00750.x. 20883352

[6] Ferrer-Blasco T. , Montés-Micó R. , Cerviño A. , Alfonso J. F. , and González-Méijome J. M. , Stereoacuity After Refractive Lens Exchange With Acrysof Restor Intraocular Lens Implantation, Journal of Refractive Surgery. (2009) 25, no. 11, 1000–1004, 10.3928/1081597x-20091016-05. 19921768

[7] Vlaskamp B. N. , Yoon G. , and Banks M. S. , Human Stereopsis Is Not Limited by the Optics of the Well-Focused Eye, Journal of Neuroscience. (2011) 31, no. 27, 9814–9818, 10.1523/jneurosci.0980-11.2011. 21734272 PMC3158004

[8] Marella B. L. , Vaddavalli P. K. , Reddy J. C. , Conway M. L. , Suttle C. M. , and Bharadwaj S. R. , Interocular Contrast Balancing Partially Improves Stereoacuity in Keratoconus, Optometry and Vision Science. (2023) 100, no. 4, 239–247, 10.1097/opx.0000000000002001. 36856557

[9] Antunes-Foschini R. M. S. , Coutinho J. , Rocha E. M. , and Bicas H. E. A. , Oculomotor Status, Binocular Vision, and Stereoacuity in a Series of Keratoconus Subjects, Investigative Ophthalmology & Visual Science. (2018) 59, no. 5, 1869–1877, 10.1167/iovs.17-23484. 29677347

[10] Khan N. , Zaka-ur-Rab S. , Ashraf M. , and Mishra A. , Comparison of Stereoacuity in Patients of Anisometropia, Isometropia and Emmetropia, Indian Journal of Ophthalmology. (2022) 70, no. 12, 4405–4409, 10.4103/ijo.ijo_658_22. 36453354 PMC9940589

[11] Burgess S. , Kousha O. , Khalil M. , Gilmour C. , MacEwen C. J. , and Gillan S. N. , Impact of Stereoacuity on Simulated Cataract Surgery Ability, Eye. (2021) 35, no. 11, 3116–3122, 10.1038/s41433-020-01346-4. 33469126 PMC8526697

[12] Dutton J. , Watkins A. , Henderson J. et al., Influence of Stereopsis on the Ability to Perform Simulated Microsurgery, Journal of Cataract & Refractive Surgery. (2020) 46, no. 4, 549–554, 10.1097/j.jcrs.0000000000000090. 32271521

[13] Gietzelt C. , Datta R. , Busshoff J. , Bruns T. , Wahba R. , and Hedergott A. , The Influence of Stereoscopic Vision on Surgical Performance in Minimal Invasive Surgery-A Substudy of the IDOSP-Study (Influence of 3D-Vs. 4 K-Display Systems on Surgical Performance in Minimal Invasive Surgery), Langenbeck’s Archives of Surgery. (2022) 407, no. 7, 3065–3074. 10.1007/s00423-022-02608-3 PMC9640404 35869334

[14] Lee S. Y. and Isenberg S. J. , The Relationship Between Stereopsis and Visual Acuity After Occlusion Therapy for Amblyopia, Ophthalmology. (2003) 110, no. 11, 2088–2092, 10.1016/s0161-6420(03)00865-0. 14597513

[15] Levi D. M. , Knill D. C. , and Bavelier D. , Stereopsis and Amblyopia: A Mini-Review, Vision Research. (2015) 114, 17–30, 10.1016/j.visres.2015.01.002. 25637854 PMC4519435

[16] Peyman A. , Pourazizi M. , Akhlaghi M. , Feizi A. , Rahimi A. , and Soltani E. , Stereopsis After Corneal Refractive Surgeries: A Systematic Review and Meta-Analysis, International Ophthalmology. (2022) 42, no. 7, 2273–2288, 10.1007/s10792-021-02201-5. 35041131

[17] Fan R. , Chan T. C. , Prakash G. , and Jhanji V. , Applications of Corneal Topography and Tomography: A Review, Clinical and Experimental Ophthalmology. (2018) 46, no. 2, 133–146, 10.1111/ceo.13136. 29266624

[18] Ghemame M. , Charpentier P. , and Mouriaux F. , Corneal Topography in Clinical Practice, Journal Français d’Ophtalmologie. (2019) 42, no. 10, e439–e451, 10.1016/j.jfo.2019.09.001. 31727328

[19] Mortazavi S. A. , Fazel F. , Radmanesh P. , Peyman A. , and Pourazizi M. , Wavefront-Guided Photorefractive Keratectomy With and Without Iris Registration: Comparison of Astigmatic Correction, Lasers in Medical Science. (2021) 36, no. 1, 75–81, 10.1007/s10103-020-03010-5. 32297251

[20] Peyman A. , Bazukar M. , Narimani T. , Mirmohammadkhani M. , and Pourazizi M. , Ocular Surface Microbial Flora and Photorefractive Keratectomy, Journal Français d’Ophtalmologie. (2022) 2022, 10.1155/2022/5029064. PMC9159841 35663519

[21] Razmjoo H. , Mikaniki M. , Peyman A. , Mikaniki E. , Abounoori M. , and Pourazizi M. , Clinical Efficacy of Fluorometholone Versus Loteprednol Eye Drops After Photorefractive Keratectomy: A Triple-Blinded Randomized Controlled Trial, European Journal of Ophthalmology. (2023) 33, no. 1, 595–601, 10.1177/11206721221106142. 35656757

[22] Grosvenor T. and Grosvenor T. P. , Primary Care Optometry, 2007, Butterworth-Heinemann/Elsevier.

[23] Koctekin B. , Coban D. T. , Ozen M. et al., Investigation of Relationship Between Colour Discrimination Ability and Stereoscopic Acuity Using Farnsworth Munsell 100 Hue Test and Stereo Tests, Canadian Journal of Ophthalmology. (2020) 55, no. 2, 131–136, 10.1016/j.jcjo.2019.07.013. 31712007

[24] Gouda M. A. , Common Pitfalls in Reporting the Use of SPSS Software, Medical Principles and Practice. (2015) 24, no. 3, 10.1159/000381953. PMC5588246 25895435

[25] Avila M. Y. and Vivas P. R. , Visual Outcomes in Hyperopic Myopic and Emmetropic Patients With Customized Aspheric Ablation (Q Factor) and Micro-Monovision, International Ophthalmology. (2021) 41, no. 6, 2179–2185, 10.1007/s10792-021-01775-4. 33725268

[26] Cuesta J. R. , Anera R. G. , Jiménez R. , and Salas C. , Impact of Interocular Differences in Corneal Asphericity on Binocular Summation, American Journal of Ophthalmology. (2003) 135, no. 3, 279–284. 12614742 10.1016/s0002-9394(02)01968-2

[27] Calossi A. , Corneal Asphericity and Spherical Aberration, Journal of Refractive Surgery. (2007) 23, no. 5, 505–514, 10.3928/1081-597x-20070501-15. 17523514

[28] Wu Y. , Wang S. , Wang G. , Zhao S. , Wei R. , and Huang Y. , Corneal Asphericity and Higher-Order Aberrations After FS-LASIK and Trans-PRK for Myopia, J Ophthalmol. (2021) 2021, 3765046–3765048, 10.1155/2021/3765046. 34912576 PMC8668292

[29] Devi P. , Kumar P. , Marella B. L. , and Bharadwaj S. R. , Impact of Degraded Optics on Monocular and Binocular Vision: Lessons From Recent Advances in Highly-Aberrated Eyes, Seminars in Ophthalmology. (2022) 37, no. 7-8, 869–886, 10.1080/08820538.2022.2094711. 35786147

[30] Sabesan R. , Zheleznyak L. , and Yoon G. , Binocular Visual Performance and Summation After Correcting Higher Order Aberrations, Biomedical Optics Express. (2012) 3, no. 12, 3176–3189, 10.1364/boe.3.003176. 23243568 PMC3521316

[31] Schwarz C. , Manzanera S. , and Artal P. , Binocular Visual Performance With Aberration Correction as a Function of Light Level, Journal of Vision. (2014) 14, no. 14, 10.1167/14.14.6. 25515764

[32] Jiménez J. R. , Castro J. J. , Jiménez R. , and Hita E. , Interocular Differences in Higher-Order Aberrations on Binocular Visual Performance, Optometry and Vision Science. (2008) 85, no. 3, 174–179, 10.1097/opx.0b013e31816445a7. 18317332

[33] Kang J. , Xiao F. , Zhao J. et al., Effects of Higher-Order Aberration Correction on Stereopsis at Different Viewing Durations, Journal of Biomedical Optics. (2015) 20, no. 7, 10.1117/1.jbo.20.7.075005. 26172611

[34] Ghoreishi M. , Peyman A. , Koosha N. , Golabchi K. , and Pourazizi M. , Topography-Guided Transepithelial Photorefractive Keratectomy to Correct Irregular Refractive Errors After Radial Keratotomy, Journal of Cataract & Refractive Surgery. (2018) 44, no. 3, 274–279, 10.1016/j.jcrs.2017.12.015. 29610024

[35] Christiansen S. M. , Neuffer M. C. , Sikder S. , Semnani R. T. , and Moshirfar M. , The Effect of Preoperative Keratometry on Visual Outcomes After Moderate Myopic LASIK, Clinical Ophthalmology. (2012) 6, 459–464, 10.2147/opth.s28808. 22536037 PMC3334209

[36] Kingston A. C. and Cox I. G. , Population Spherical Aberration: Associations With Ametropia, Age, Corneal Curvature, and Image Quality, Clinical Ophthalmology. (2013) 7, 933–938, 10.2147/opth.s44056. 23723685 PMC3666881

[37] Koosha N. , Fathian A. , Peyman A. , Nourbakhsh S. A. , Noorshargh P. , and Pourazizi M. , Combined Simultaneous Photorefractive Keratectomy and Collagen Cross-Linking in Keratoconus Suspect Patients, Journal Français d’Ophtalmologie. (2023) 46, no. 8, 921–928, 10.1016/j.jfo.2022.11.029. 37085363

[38] Peyman A. , Feizi A. , Ganjalikhani-Hakemi M. , Hosseini-Nasab F. , and Pourazizi M. , Outcome of Corneal Collagen Cross-Linking in Keratoconus: Introducing the Predictive Factors, Journal of Current Ophthalmology. (2020) 32, no. 1, 19–25, 10.4103/joco.joco_48_20. 32510009 PMC7265260

[39] Randleman J. B. , Trattler W. B. , and Stulting R. D. , Validation of the Ectasia Risk Score System for Preoperative Laser in Situ Keratomileusis Screening, American Journal of Ophthalmology. (2008) 145, no. 5, 813–818, 10.1016/j.ajo.2007.12.033. 18328998 PMC3748728

[40] Santhiago M. R. , Percent Tissue Altered and Corneal Ectasia, Current Opinion in Ophthalmology. (2016) 27, no. 4, 311–315, 10.1097/icu.0000000000000276. 27096376

[41] Dodgson N. , Variation and Extrema of Human Interpupillary Distance, 2020, Open Access Te Herenga Waka-Victoria University of Wellington.

[42] Eom Y. , Song J. S. , Ahn S. E. et al., Effects of Interpupillary Distance on Stereoacuity: the Frisby Davis Distance Stereotest Versus a 3-Dimensional Distance Stereotest, Japanese Journal of Ophthalmology. (2013) 57, no. 5, 486–492, 10.1007/s10384-013-0253-9. 23828094

[43] Remón L. , Monsoriu J. A. , and Furlan W. D. , Influence of Different Types of Astigmatism on Visual Acuity, Journal of Optics. (2017) 10, no. 3, 141–148, 10.1016/j.optom.2016.07.003. PMC5484781 27639497

[44] Wolffsohn J. S. , Bhogal G. , and Shah S. , Effect of Uncorrected Astigmatism on Vision, Journal of Cataract & Refractive Surgery. (2011) 37, no. 3, 454–460, 10.1016/j.jcrs.2010.09.022. 21333869

[45] Peyman A. , Ghoreishi M. , Babaei L. , Noorshargh P. , Forouhari A. , and Pourazizi M. , Transepithelial Versus Epithelium-Off Photorefractive Keratectomy in High Compound Myopic Astigmatism: A Contralateral Eye Study, Journal of Refractive Surgery. (2024) 40, no. 12, e956–e965, 10.3928/1081597x-20241021-03. 39656257

[46] Al-Qahtani H. and Al-Debasi H. , The Effects of Experimentally Induced Graded Monocular and Binocular Astigmatism on Near Stereoacuity, Saudi Journal of Ophthalmology. (2018) 32, no. 4, 275–279, 10.1016/j.sjopt.2018.09.001. 30581296 PMC6300757

[47] Kulkarni V. , Puthran N. , and Gagal B. , Correlation Between Stereoacuity and Experimentally Induced Graded Monocular and Binocular Astigmatism, Journal of Clinical and Diagnostic Research. (2016) 10, no. 5, Nc14–Nc17. 27437256 10.7860/JCDR/2016/17341.7873 PMC4948432

